# Is the Risk of Preterm Birth and Low Birth Weight Affected by the Use of Antidepressant Agents during Pregnancy? A Population-Based Investigation

**DOI:** 10.1371/journal.pone.0168115

**Published:** 2016-12-15

**Authors:** Anna Cantarutti, Luca Merlino, Emiliano Monzani, Carlo Giaquinto, Giovanni Corrao

**Affiliations:** 1 Department of Statistics and Quantitative Methods, Division of Biostatistics, Epidemiology and Public Health, Laboratory of Healthcare Research and Pharmacoepidemiology, University of Milano-Bicocca, Milan, Italy; 2 Operative Unit of Territorial Health Services, Region of Lombardy, Milan, Italy; 3 Department of Mental Health, Cà Granda Niguarda Hospital, Milan, Italy; 4 Department of Women's and Children's Health, University of Padova, Padova, Italy; Centre Hospitalier Universitaire Vaudois, FRANCE

## Abstract

**Background:**

Untreated depression during pregnancy increases the risk of morbidity and mortality in the mother and child. Therefore, specific treatments are required for this population.

**Objective:**

The study aimed to investigating the effect of antidepressant medication used during pregnancy with reference to the risk of preterm birth (PTB) and low birth weight (LBW).

**Methods:**

A population-based study was carried out with data provided by the healthcare utilization database of Lombardy, an Italian region with about ten million inhabitants. The study included 384,673 births from 2005 to 2010. Maternal use of antidepressants before and during pregnancy was investigated. Log-binomial regression was used to estimate the association between the use of antidepressants during pregnancy, compared to the non-use or use just before pregnancy, and the prevalence ratio of PTB and LBW.

**Results:**

Women who used antidepressants during pregnancy had a 20% (95% CI: 10–40%) increased prevalence of both PTB and LBW compared to those who never used antidepressants. There was no evidence that women who used antidepressants during pregnancy had a higher prevalence of the considered outcomes compared to women who used antidepressants before pregnancy, but stopped during pregnancy. Such findings were confirmed by considering separately the effects of SSRIs and other antidepressants together.

**Conclusions:**

Our findings suggest that depression in itself, rather than antidepressant medication, might be implicated in the causal pathway of PTB and LBW.

## Introduction

Depression affects up to 13% of women in reproductive age [1]. Untreated antenatal depression has been found to correlate with poor self-care during pregnancy, postpartum depression, impaired maternal–infant attachment and delays in infant development [2,3], so that antidepressant medication may be required for the effective treatment of maternal depression [4,5].

Antidepressant drugs, developed since 1950s to treat depressive symptoms, are nowadays widely available with several treatment options. Tricyclic Antidepressants and Selective Serotonin Reuptake Inhibitors (SSRIs), are the most commonly prescribed antidepressants. Despite their similar effectiveness, however, SSRIs have in part replaced Tricyclic Antidepressants due to better tolerability [6].

Several studies over the past two decades investigated the relationship between the use of antidepressants in pregnancy and the risk of adverse perinatal and birth outcomes [7]. Exposure in utero to antidepressants has been associated with low birth weight and preterm delivery [8–11]. The biological mechanisms explaining the relationship between using antidepressants during pregnancy and delivery outcomes are not entirely known, although some assumptions have been postulated [12–17]. However, as maternal depression may be related to unhealthy behaviors—such as smoking and poor attendance of obstetric care [18–20]—it is still unclear whether the observed adverse perinatal outcomes may be due to direct drug actions or to depression itself [2, 21–23].

The purpose of this population-based study was to investigate the effect of the use of antidepressant medication during pregnancy with reference to the risk of preterm birth (PTB) and low birth weight (LBW).

## Methods

### Setting

The data used for this study were provided by the healthcare utilization databases of Lombardy, an Italian Region with about 16% of the country’s population (almost ten million inhabitants). In Italy, the population is covered by the National Health Service (NHS), which in Lombardy has been associated, since 1997, with an automated system of databases to collect a variety of information including: (1) an archive of those benefitting from the Regional Health Service (practically coinciding with the whole resident population), reporting demographic and administrative data; (2) a database concerning diagnoses at discharge from Italian public or private hospitals; (3) a database concerning outpatient drug prescriptions reimbursed by the NHS and delivered by pharmacies in Lombardy; and (4) a database reporting the Certificates of Delivery Assistance (i.e., the so called CeDAP) providing detailed information on the mother’s socioeconomic traits, as well as medical information on the pregnancy, childbirth, and child presentation at delivery. The linking of records among databases, owing to a unique code in all databases, allowed to identify a large and unselected birth cohort and to reconstruct relevant traits and care pathways of mothers and newborns.

### Cohort selection

**Fig 1:**
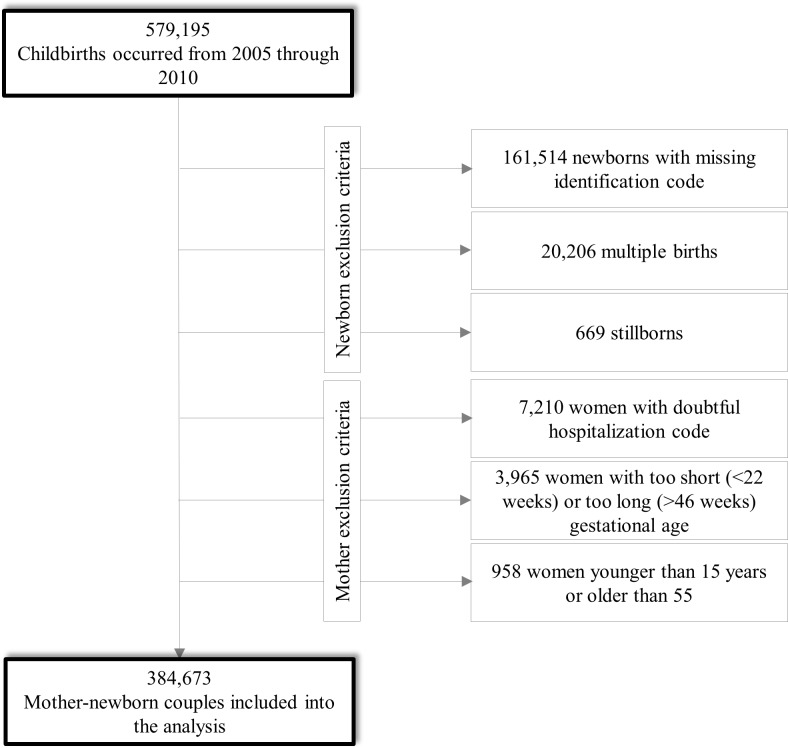
Flow-chart of inclusion and exclusion criteria.

The 579,195 childbirths of women resident in Lombardy from 2005 to 2010 were selected from the CeDAP database. Among these, 182,389 (31.5%) were excluded because the newborn (i) had no identification code (161,514), (ii) was part of multiple birth (20,206), or (iii) was stillborn (669). Further 12,133 records (2.1%) were excluded because the mother (i) had a hospital admission ICD-9 code different from the one expected for childbirth (7,210), (ii) had a too short (<22 weeks) or too long (>46 weeks) gestational age (3,965), or (iii) was under 15 years of age or above 55 years of age (958). The final study population therefore consisted of 384,673 mother-newborn couples (**Fig 1**).

### Use of antidepressants and other features concerning the mother

All prescriptions of antidepressant medication dispensed to the women considered during the period of observation, starting from the date corresponding to 9 months before the expected date of conception and stopping at the date of childbirth, were identified. Mothers were thus classified in the following mutually exclusive categories: (i) non-users, if antidepressants were not dispensed during the entire period of observation; (ii) users just before conception, if at least an antidepressant was dispensed in the 9 months before, but not during, pregnancy; and (iii) users during pregnancy, otherwise.

Maternal traits, including age at delivery, nationality, marital status, education, employment, previous miscarriages and parity, and health conditions, including diabetes, preeclampsia, dyslipidaemia and hypertension, were identified through CeDAPs.

### Neonatal outcomes

Two outcomes were considered: “preterm birth” (less than 37 weeks’ gestation [24]), and “low birth weight” (less than 2,500 grams [25]) identified from CeDAPs.

### Statistical analysis

Chi-squared, or its version for the trend, was used when appropriate for testing differences or trends in maternal socio-demographic and clinical features according to maternal use of antidepressants.

The log-binomial regression model was separately fitted to estimate the prevalence ratio (PR), and the 95% confidence interval (95% CI) of each neonatal outcome associated with the use of antidepressants during pregnancy compared to non-use or use just before pregnancy, as well as of use just before pregnancy compared to non-use. Estimates were adjusted for the maternal traits and health conditions listed above. A generalized estimating equation was used to account for the potential correlation of women contributing with more than one birth during the considered period. Besides the effect of antidepressants as a whole, the separate effects of agents belonging to the class of SSRIs and to other antidepressants was investigated.

Data on maternal characteristics were sometimes missing. Indeed, missing data ranged from 1% for previous miscarriages to 13% for marital status. Restricting analyses to the subset of women with all the data observed would have resulted in a significant loss of information and possibly biased estimations. With the aim to generate appropriate values of missing data for those women with missing covariates, the three-phase iterative procedure known as the fully conditional specification (FCS) was used [26,27]. First of all, the FCS method was implemented to generate 10 complete data sets. Secondly, the log-binomial model was separately fitted to the 10 complete data sets using the GENMOD procedure. Finally, the MIANALYZE procedure was used to combine the coefficient estimates (and estimations of their variances) from the 10 log-binomial analyses, in order to obtain valid statistical inferences about the model coefficients that take within and between variances into account.

All analyses were performed using the Statistical Analysis System Software (version 9.4; SAS Institute, Cary, NC, USA). Statistical significance was set at the 0.05 level. All p-values were two-sided.

## Results

During the entire observation period (i.e., from 9 months before starting pregnancy until childbirth), antidepressant medication were dispensed at least once to 9,843 women among those the 384,673 included (prevalence: 2.6%). Most women stopped using antidepressants during pregnancy (users just before pregnancy: 6,548 women), while 3,295 mothers kept on following the therapy during pregnancy (users during pregnancy). **Table 1** shows that, compared to both non-users and users just before pregnancy, women who used antidepressants during pregnancy were older, with lower education, and more often were Italian, unmarried, employed, and suffered from the considered medical conditions. Previous pregnancies were significant predictors of the use of antidepressants during pregnancy compared to non-use.

**Table 1:** Selected characteristics of the 384,673 mothers considered in the study according to their use of antidepressants before conception or during pregnancy. Italy, Region of Lombardy, 2005–2010.

	Use of antidepressants			p-value ^2^		
	Never (A) N = 374,830	Just before pregnancy (B) N = 6,548	During pregnancy (C) N = 3,295			
				A vs. C	B vs. C	
Age at delivery						
≤ 25 years	13.3%	8.2%	8.2%	<0.0001		<0.0001
26–34 years	56.9%	55.9%	48.9%			
>34 years	29.8%	35.9%	41.9%			
Nationality						
Italy	74.6%	85.6%	86.5%	<0.0001		0.0004
Other	25.4%	14.4%	13.5%			
Marital status						
Married	77.0%	73.2%	72.2%	<0.0001		0.0006
Unmarried	23.0%	26.8%	27.8%			
Education ^1^						
Low	31.6%	34.3%	34.4%	<0.0001		0.0037
Intermediate	45.4%	47.1%	46.5%			
High	23.0%	18.6%	19.1%			
Employment						
Employed	70.4%	74.1%	71.7%	<0.0001		<0.0001
Unemployed	29.6%	25.9%	28.3%			
Previous miscarriages						
None	83.6%	82.6%	83.0%	0.0049		0.1207
One or more	16.4%	17.4%	17.0%			
Parity						
Nulliparous	55.3%	52.6%	53.0%	<0.0001		0.3404
Multiparous	44.7%	47.3%	47.0%			
Medical conditions						
Diabetes	5.0%	6.3%	7.2%	<0.0001	<0.0001	
Hypertension	9.4%	16.2%	18.2%	<0.0001	<0.0001	
Dyslipidaemia	2.1%	3.2%	4.4%	<0.0001	<0.0001	
Preeclampsia	1.2%	1.3%	1.5%	0.1510	0.0289	

^1^ Number of years of formal education completed categorized as 8 or fewer (low), from 9 to 13 (intermediate) and or 14 or more (high)

^2^ According to the chi-square test, or its version for the trend (age and education)

**Fig 2:**
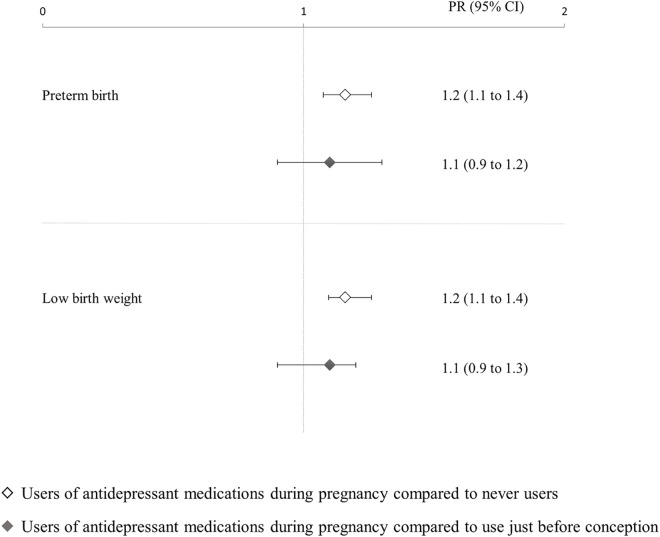
Adjusted prevalence ratios (and 95% confidence intervals) of selected outcomes associated with the use of antidepressants during pregnancy, compared to the non-use as well as to the use just before pregnancy. Prevalence ratio, and 95% confidence interval, estimated with log-binomial regression. Estimates are adjusted for maternal age, nationality, marital status, education, employment, previous miscarriages, parity, and medical conditions.

Out of the 384,673 newborns considered in this study, 20,060 (5.2%) and 19,527 (5.1%) had preterm birth and low birth weight, respectively. **Fig 2** shows that mothers who used antidepressants during pregnancy had significant higher prevalence of preterm birth and low birth weight with respect to those who never used antidepressants, but not to those who used antidepressants just before pregnancy. Statistical evidence of higher prevalence of both outcomes among women who stopped using depressant before pregnancy with respect to those who never used them was also found, being the adjusted PRs (and corresponding 95% CI) 1.1 (1.0 to 1.2) and 1.1 (1.0 to 1.3) for preterm birth and low birth weight respectively.

The prevalence of preterm birth among women who used either SSRIs or other antidepressants during pregnancy, as well as the prevalence of low birth weight among women who used SSRIs during pregnancy, were significantly higher with respect to mothers who never used antidepressants, but did not differ from those who used antidepressants just before pregnancy (**Table 2**). Finally, likely due to inadequate power for pointing out the effect of other antidepressants, women using them during pregnancy and those who never used antidepressants did not show significant difference in prevalence of low birth weight.

**Table 2:** Adjusted prevalence ratios (and 95% confidence intervals) of selected outcomes associated with dispensing selective serotonin reuptake inhibitors (SSRIs) or other antidepressant medication during pregnancy compared to non-users and users just before pregnancy. Italy, Region of Lombardy, 2005–2010.

Neonatal outcome	Comparator	SSRI^1^	Other antidepressants^1^
Preterm birth	Non-users	1.2 (1.1 to 1.4)	1.3 (1.1 to 1.5)
	Users just before pregnancy	1.1 (0.9 to 1.2)	1.0 (0.8 to 1.3)
Low birth weight	Non-users	1.3 (1.1 to 1.5)	1.3 (0.9 to 1.7)
	Users just before pregnancy	1.1 (0.9 to 1.3)	1.1 (0.8 to 1.5)

^1^Prevalence ratio, and 95% confidence interval, estimated with log-binomial regression. Estimates are adjusted for maternal age, nationality, marital status, education, employment, previous miscarriages, parity, and medical conditions

## Discussion

Our large population-based study found that women who used antidepressants during pregnancy had a 20% (95% CI: 10–40%) increased prevalence of both preterm birth and low birth weight compared to those who never used antidepressants during the entire period of observation (i.e., from 9 months before pregnancy until childbirth). Such evidence was confirmed by considering separately the effects of SSRIs and other antidepressants together.

These findings confirm and extend the results of (i) meta-analyses showing that prenatal exposure to antidepressant medication as a whole [8,9], as well as to SSRIs [28], reduces gestational age and birth weight; and (ii) observational studies reporting an association between prenatal use of antidepressants and risks for premature delivery [15,29–33] and low birth weight [32,34,35].

At least two possible explanations are conceivable with our findings. Firstly, the safety of antidepressants on foetal health might be the mechanistic key explaining the higher prevalence of adverse neonatal outcomes among drug users. Although the biological mechanisms are not entirely known, several theories have been postulated on this issue. Antidepressants, mainly SSRIs, pass the placenta barrier increasing the placental secretion of corticotrophin-releasing hormone resulting in an increased activity within the gestational cortisol system [11]. Furthermore, fluoxetine reduces maternal appetite and weight gain causing low birth weight [13, 14]. Moreover, the use of SSRIs alters the 5-TH levels increased risk of intrauterine growth retardation and preterm delivery by impairing placental blood flow [15]. It is also reported that women using antidepressants had higher saliva estriol levels compared to non-users [16] and elevated levels of estriol have been associated with preterm birth [17]. Secondly, antidepressant medication are prescribed to treat depression so that the observed associations could be explained by the residual depressive symptoms. We tried to account for confounding indications by constraining women who took antidepressants during pregnancy with those who interrupted their use during pregnancy. Interestingly, our study did not offer statistical evidence that the considered outcomes differed between using medication before or during pregnancy. In addition, higher prevalence of preterm birth and low birth weight among newborns from women who used antidepressants just before pregnancy than from those who never used them was observed. All these findings taken together suggest that, at least in our setting, depression in itself, rather than antidepressant medication, might be implicated in the causal pathway of these outcomes [36]. The mechanism by which depression may exert its action on the considered neonatal outcomes might be mediated by the presence of epiphenomena, e.g., smoking, alcohol drinking, and other unhealthy behaviours, such as poor attendance to obstetric care [18–20].

Our study has a number of potential limitations. First of all, the exclusion of mother-newborn pairs lacking identification codes could mainly affect less healthy women. Second, the implicit exclusion from our analysis of spontaneous and elective pregnancy terminations affects the possibility for outcomes potentially due to drug foetal-exposure to be selectively excluded. Third, a main limitation in using dispensing data relates to whether or not the medicine was consumed, or consumed as directed, and there is no information in this study for either of these aspects [37]. Fourth, privacy concerns prevented us to assess the validity of the information recorded in the Certificates of Delivery Assistance, as well as the diagnostic data from hospital charts. Fifth, we did not assess when antidepressants were used during pregnancy, a datum which would have provided information concerning possible heterogeneity in outcome risks during the observation period. There are two reasons for the lack of assessment: dispensation data certainly does not correspond to use data; and power considerations did not allow the assessment of rarer exposures than those observed. Finally, the lack of data on important factors—such as smoking, alcohol and illicit drug use—may further contribute to some unavoidable source of systematic uncertainty.

Despite these limitations, our data on drug utilization patterns in the real-world setting offer evidence that the prevalence of preterm birth and low birth weight is increased in pregnant women who use antidepressants during pregnancy compared to pregnant women who never use antidepressants. However, rather than a direct action of these agents, our findings suggest that depression in itself may explain the observed adverse neonatal outcomes, possibly due to the effect of maternal unhealthy behaviours, such as smoking, alcohol abuse, unhealthy diet, and poor attendance to obstetric care. Much more research is needed to better understand risks and benefits of therapeutic strategies for depression care during pregnancy.

## References

[1] Gavin NI, Gaynes BN, Lohr KN, Meltzer-Brody S, Gartlehner G, Swinson T. Perinatal depression: a systematic review of prevalence and incidence. Obstet Gynecol 2005;106:1071–83 10.1097/01.AOG.0000183597.31630.db 16260528

[2] Grote NK, Bridge JA, Gavin AR, Melville JL, Iyengar S, Katon WJ. A meta-analysis of depression during pregnancy and the risk of preterm birth, low birth weight, and intrauterine growth restriction. Arch Gen Psychiatry 2010;67(10):1012–24 10.1001/archgenpsychiatry.2010.111 20921117 PMC3025772

[3] Field T. Postpartum depression effects on early interactions, parenting, and safety practices: a review. Infant Behav Dev 2010;33:1–6 10.1016/j.infbeh.2009.10.005 19962196 PMC2819576

[4] Yonkers K, Vigod S, Ross L. Diagnosis, pathophysiology, and management of mood disorders in pregnant and postpartum women. Obstet Gynecol 2011;117:961–77 10.1097/AOG.0b013e31821187a7 21422871

[5] APA. Practice guideline for the treatment of patients with major depressive disorder 3rd ed.; American Psychiatric Association 2010 [cited 10/21/2012]; Available from: http://psychiatryonline.org/content.aspx?bookid=28&sectionid=1667485

[6] Tata LJ, West J, Smith C, Farrington P, Card T, Smeeth L, et al General population based study of the impact of tricyclic and selective serotonin reuptake inhibitor antidepressants on the risk of acute myocardial infarction. Heart 2005;91:465–71 10.1136/hrt.2004.037457 15772201 PMC1768803

[7] Udechuku A, Nguyen T, Hill R, Szego K. Antidepressants in pregnancy: a systematic review. Aust N Z J Psychiatry 2010;44:978–96 10.3109/00048674.2010.507543 21034181

[8] Ross Le, Grigoriadis S, Mamisashvili L, Vonderporten EH, Roerecke M, Rehm J, et al Selected pregnancy and delivery outcomes after exposure to antidepressant medication: a systematic review and meta-analysis. JAMA Psychiatry 2013;70:436–43 10.1001/jamapsychiatry.2013.684 23446732

[9] Huang H, Coleman S, Bridge JA, Yonkers K, Katon W. A meta-analysis of the relationship between antidepressant use in pregnancy and the risk of preterm birth and low birth weight. Gen Hosp Psychiatry 2014;36:13–8 10.1016/j.genhosppsych.2013.08.002 24094568 PMC3877723

[10] Simon GE, Cunningham ML, Davis RL. Outcomes of prenatal antidepressant exposure. Am J Psychiatry 2002;159:2055–2061 10.1176/appi.ajp.159.12.2055 12450956

[11] Ramos E, St-Andre M, Berard A. Association between antidepressant use during pregnancy and infants born small for gestational age. Can J Psychiatry 2010;55:643–652 20964943

[12] Rampono J, Simmer K, Ilett KF, Hackett LP, Doherty DA, Elliot R, et al Placental transfer of SSRI and SNRI antidepressants and effects on the neonate. Pharmaco-psychiatry 2009; 42:95–100 10.1055/s-0028-1103296 19452377

[13] Yonkers K.A., Norwitz E. R., Smith M. V., Lockwood C. J., Gotman N., Luchansky E., et al Depression and serotonin reuptake inhibitor treatment as risk factors for preterm birth. Epdemiology 2012;23:677–685 10.1097/EDE.0b013e31825838e9 PMC3415566 22627901

[14] Akerud H, Kaihola H, Pawluski JL, Skalkidou A, Högberg U, Sundström-Poromaa I. The effects of maternal depression and maternal selective serotonin reuptake inhibitor exposure on offspring. Front Cell Neurosci 2013;7:73 10.3389/fncel.2013.00073 23734100 PMC3659337

[15] Wen SW, Yang Q, Garner P, Fraser W, Olatunbosun O, Nimrod C, et al Selective serotonin reuptake inhibitors and adverse pregnancy outcomes. Am J Obstet Gynecol 2006;194:961–6 10.1016/j.ajog.2006.02.019 16580283

[16] Suri R, Hellemann G, Cohen L, Aquino A, Altshuler L. Saliva estriol levels in women with and without prenatal antidepressant for treatment. Biol Psychiatry. 2008;64(6):533–537 10.1016/j.biopsych.2008.04.015 18495086 PMC2562039

[17] McGregor JA, Jackson GM, Lachelin GC, Goodwin TM, Artal R, Hastings C, et al Salivary estriol as risk assessment for preterm labor: a prospective trial. Am J Obstet Gynecol. 1995;173(4):1337–42 7485350 10.1016/0002-9378(95)91383-1

[18] Cinciripini PM, Blalock JA, Minnix JA, Robinson JD, Brown VL, Lam C, et al Effects of an intensive depression-focused intervention for smoking cessation in pregnancy. J Consult Clin Psychol 2010;78:44–54 10.1037/a0018168 20099949 PMC2881321

[19] Källén K. The impact of maternal smoking during pregnancy on delivery outcome. Eur J Public Health 2001;11:329–33 11582615 10.1093/eurpub/11.3.329

[20] Alwan S, Reefhuis J, Rasmussen SA, Friedman JM. Patterns of antidepressant medication use among pregnant women in a United States population. J Clin Pharmacol 2011;51:264–270 10.1177/0091270010373928 20663997

[21] El Marroun H, Jaddoe VW, Hudziak JJ, Roza SJ, Steegers EA, Hofman A, et al Maternal use of selective serotonin reuptake inhibitors, fetal growth, and risk of adverse birth outcomes. Arch Gen Psychiatry 2012;69:706–14 10.1001/archgenpsychiatry.2011.2333 22393202

[22] Preti A, Cardascia L, Zen T, Pellizzari P, Marchetti M, Favaretto G, et al Obstetric complications in patients with depression—a population-based case-control study. J Affect Disord 2000; 61:101–6 11099747 10.1016/s0165-0327(99)00185-8

[23] Barker ED, Copeland W, Maughan B, Jaffee SR, Uher R. Relative impact of maternal depression and associated risk factors on offspring psychopathology. Br J Psychiatry 2012; 200:124–9 10.1192/bjp.bp.111.092346 22241929 PMC3567912

[24] Lawn JE, Gravett MG, Nunes TM, Rubens CE, Stanton C; GAPPS Review Group. Global report on preterm birth and stillbirth (1 of 7): definitions, description of the burden and opportunities to improve data. BMC Pregnancy Childbirth 2010;10 Suppl 1:S1 20233382 10.1186/1471-2393-10-S1-S1 PMC2841772

[25] Valero de Bernabé J, Soriano T, Albaladejo R, Juarranz M, Calle ME, Martínez D, et al Risk factor for low birth weight: a review. Eur J Obstet Gynecol 2004;116:3–15 10.1016/j.ejogrb.2004.03.007 15294360

[26] Magnus MC, Stigum H, Håberg SE, Nafstad P, London SJ, Nystad W. Peak weight and height velocity to age 36 months and asthma development: The Norwegian Mother and Child Cohort Study. PLoS One. 2015;10(1):e0116362 10.1371/journal.pone.0116362 25635872 PMC4312021

[27] Verret-Chalifour J, Giguère Y, Forest JC, Croteau J, Zhang P, Marc I. Breastfeeding initiation: impact of obesity in a large Canadian perinatal cohort study. PLoS One. 2015 2 6;10(2):e0117512 10.1371/journal.pone.0117512 25659144 PMC4320116

[28] Lattimore KA, Donn SM, Kaciroti N, Kemper AR, Neal CR Jr, Vazquez DM. Selective serotonin reuptake inhibitor (SSRI) use during pregnancy and effects on the fetus and newborn: a meta-analysis. J Perinatol 2005;25:595–604 10.1038/sj.jp.7211352 16015372

[29] Davis RL, Rubanowice D, McPhillips H, Raebel MA, Andrade SE, Smith D, et al Risks of congenital malformations and perinatal events among infants exposed to antidepressant medications during pregnancy. Pharmacoepidemiol Drug Saf 2007;16:1086–94 10.1002/pds.1462 17729378

[30] Ferreira E, Carceller AM, Agogue´ C, Martin BZ, St-Andre´ M, Francoeur D, et al Effects of selective serotonin reuptake inhibitors and venlafaxine during pregnancy in term and preterm neonates. Pediatrics 2007;119:52–9 10.1542/peds.2006-2133 17200271

[31] Simon GE, Cunningham ML, Davis RL. Outcomes of prenatal antidepressant exposure. Am J Psychiatry 2002;159:2055–61 10.1176/appi.ajp.159.12.2055 12450956

[32] Kallen B. Neonate characteristics after maternal use of antidepressants in late pregnancy. Arch Pediatr Adolesc Med 2004;158:312–6 10.1001/archpedi.158.4.312 15066868

[33] Maschi S, Clavenna A, Campi R, Schiavetti B, Bernat M, Bonati M. Neonatal outcome following pregnancy exposure to antidepressants: a prospective controlled cohort study. BJOG. 2008;115:283–9 10.1111/j.1471-0528.2007.01518.x 17903222

[34] Lund N, Pedersen LH, Henriksen TB. Selective serotonin reuptake inhibitor exposure in utero and pregnancy outcomes. Arch Pediatr Adolesc Med 2009;163:949–54 10.1001/archpediatrics.2009.164 19805715

[35] Wisner KL, Sit DK, Hanusa BH, Moses-Kolko EL, Bogen DL, Hunker DF. Major depression and antidepressant treatment: impact on pregnancy and neonatal outcomes. Am J Psychiatry 2009;166:557–66 10.1176/appi.ajp.2008.08081170 19289451 PMC4426499

[36] Jensen HM, Grøn R, Lidegaard O, Pedersen LH, Andersen PK, Kessing LV. The effects of maternal depression and use of antidepressants during pregnancy on risk of a child small for gestational age. Psychopharmacology (Berl) 2013;228(2):199–205 23455598 10.1007/s00213-013-3029-5

[37] Corrao G, Mancia G. Generating evidence from computerized healthcare utilization databases. Hypertension 2015;62:490–8 10.1161/HYPERTENSIONAHA.114.04858 25624339

